# Impact of COVID-19 Booster Vaccination on Serum Redox Homeostasis

**DOI:** 10.3390/ijms27104574

**Published:** 2026-05-20

**Authors:** Marija Vukčević, Dušan Mihajlo Spasić, Vladimir Kešelj, Lena Platanić Arizanović, Tanja Grahovac, Teodora Vidonja Uzelac, Zorana Oreščanin Dušić, Aleksandra Nikolić-Kokić, Milan Nikolić

**Affiliations:** 1 Institute for Biocides and Medical Ecology, Trebevićka 16, 11030 Belgrade, Serbia; marija.vukcevic@biocidi.org.rs; 2 Department of Pharmacology, Clinical Pharmacology and Toxicology, Faculty of Medicine, University of Belgrade, 11000 Belgrade, Serbia; dusan.spasic.dr@med.bg.ac.rs; 3 Department of Biochemistry, Faculty of Chemistry, University of Belgrade, Studentski trg 12-16, 11158 Belgrade, Serbia; vladokeselj123@gmail.com; 4 Independent Researcher, 11000 Belgrade, Serbia; lena.arizanovic@gmail.com; 5 Department of Physiology, Institute for Biological Research “Siniša Stanković”—National Institute of the Republic of Serbia, University of Belgrade, 11000 Belgrade, Serbia; tanja.grahovac@ibiss.bg.ac.rs (T.G.); teodora.vidonja@ibiss.bg.ac.rs (T.V.U.); zoranaor@ibiss.bg.ac.rs (Z.O.D.)

**Keywords:** Pfizer vaccine, Sinopharm vaccine, Sputnik V vaccine, serum, redox homeostasis

## Abstract

This study examined alterations in serum redox biomarkers before and one month after administration of the coronavirus disease 2019 (COVID-19) booster (third) doses across four vaccine regimens. A longitudinal cohort of 410 adults was analyzed following homologous Pfizer-BioNTech, Sinopharm [Vero Cell]-Inactivated, Sputnik V, or heterologous Sinopharm/Pfizer vaccination. Serum total proteins, albumin, total thiols, nitrites, ferric-reducing antioxidant power (FRAP), and 2,2-diphenyl-1-picrylhydrazyl (DPPH) radical-scavenging activity were measured, with DPPH interpreted as an ex vivo surrogate of serum radical-scavenging capacity. Additional analyses included stratification by prior severe acute respiratory syndrome coronavirus 2 (SARS-CoV-2) infection, multivariable regression, correlation analysis, effect-size estimation, and sensitivity testing. Booster vaccination was associated with modest but consistent decreases in DPPH activity, albumin, and total proteins, whereas FRAP, nitrite, and total thiol levels remained stable. This pattern supports a transient shift in antioxidant buffering capacity but, by itself, does not exclude oxidative stress, as direct oxidative damage markers were not assessed. The most pronounced changes were observed in Sinopharm-based regimens, particularly in the heterologous Sinopharm/Pfizer group. Prior SARS-CoV-2 infection did not materially alter the qualitative response pattern, whereas older age and comorbidities were associated with greater declines in DPPH activity and albumin. Overall, the findings indicate a modest, transient redox-associated response following booster-induced immune activation and suggest that host-related factors, such as age and comorbidity burden, may accentuate short-term changes in antioxidant buffering capacity.

## 1. Introduction

The coronavirus disease 2019 (COVID-19), caused by severe acute respiratory syndrome coronavirus 2 (SARS-CoV-2), has posed significant challenges to global public health and prompted extensive investigation into its immunopathogenesis and vaccine-induced responses. Beyond its direct virological effects, COVID-19 is increasingly recognised as a condition characterised by redox and inflammatory imbalances, with oxidative stress as a key mechanistic factor [1,2,3]. After primary immunisation with two vaccine doses, the immune response to SARS-CoV-2 gradually declined to levels below protective thresholds, prompting the recommendation of a third, or booster, dose. The emergence and global spread of new SARS-CoV-2 variants of concern, which require higher neutralising antibody titres for effective virus neutralisation, together with the waning humoral immune response following initial vaccination, were key factors underlying the World Health Organization recommendation for booster vaccination [4]. This recommendation was particularly emphasised for individuals with weakened immune systems, older adults, and patients with chronic diseases, who remained at higher risk of severe COVID-19 despite primary vaccination. Based on clinical data from the National Institute of Allergy and Infectious Diseases, the U.S. Food and Drug Administration authorised the use of heterologous booster vaccination, also known as the “mix-and-match” approach [5]. This policy enabled individuals who had completed a primary vaccination series with a single vaccine type (e.g., Johnson & Johnson [Ad26.COV2.S], Moderna [mRNA-1273], or Pfizer-BioNTech [BNT162b2]) to receive a booster dose from a different manufacturer, provided they met the recommended time intervals and eligibility criteria. This approach was also implemented in Serbia, particularly among individuals who initially received inactivated vaccines (e.g., Sinopharm [Vero Cell]-Inactivated COVID-19 vaccine) and subsequently received an mRNA vaccine as a booster dose [6]. Although these booster strategies enhanced protective immunity, repeated antigenic stimulation also engages metabolic and inflammatory pathways closely linked to redox regulation [7].

Oxidative stress arises when the production of reactive oxygen and nitrogen species (ROS/RNS) exceeds the neutralising capacity of antioxidant defences [8]. In SARS-CoV-2 infection, viral replication, mitochondrial dysfunction, activation of NADPH oxidases, and pro-inflammatory signalling collectively increase ROS/RNS production, resulting in redox imbalance and biomolecular damage [9,10]. Numerous studies have shown that patients with COVID-19 exhibit significant changes in redox biomarkers, including reduced thiol concentrations, increased lipid peroxidation, and decreased total antioxidant capacity, which correlate with disease severity and poor clinical outcomes [11,12]. Conversely, the redox effects of vaccination, particularly booster doses in healthy individuals, are not well characterised. Early reports indicate that vaccination may transiently alter oxidative and antioxidant parameters [13], but comprehensive longitudinal studies remain limited. Understanding how booster immunisation affects redox homeostasis is important for several reasons. Vaccine-induced immune activation can temporarily increase ROS production through respiratory burst and cytokine-mediated pathways, while adaptive mechanisms may prompt compensatory upregulation of antioxidant systems. Furthermore, baseline redox status, influenced by age, comorbidities, sex, or prior SARS-CoV-2 infection, may affect the magnitude of these responses [14]. Characterising such short-term redox changes could clarify the physiological relationship between oxidative stress and immune activation.

Importantly, redox biomarkers should not be interpreted in isolation. COVID-19 progression and outcomes are influenced by integrated inflammatory, coagulation, and metabolic networks, and combined laboratory panels that include inflammatory cell indices, D-dimer, and iron-status variables can improve risk stratification, particularly in vulnerable populations [15]. Iron metabolism is particularly relevant to redox biology because dysregulated iron handling can promote ROS generation, lipid peroxidation, and ferroptosis in severe SARS-CoV-2 infection [16]. Similarly, acute-phase responses modify circulating protein composition, including negative acute-phase proteins such as albumin, which may link immune activation to changes in protein-associated antioxidant buffering [17]. Accordingly, vaccine-related redox changes should be framed as one biochemical layer within a broader immunometabolic response, rather than as standalone evidence of systemic oxidative disease or vaccine-associated tissue injury.

In this study, we evaluated serum redox biomarkers before and one month after administration of a COVID-19 booster dose in a cohort of 410 participants across four vaccine regimens: three doses of the Pfizer-BioNTech vaccine, three doses of the Sinopharm [Vero Cell]-Inactivated vaccine, three doses of Sputnik V (SpSpSP), and two Sinopharm doses followed by a Pfizer-BioNTech booster (SSP). The analysed parameters included 2,2-diphenyl-1-picrylhydrazyl (DPPH) radical-scavenging activity, ferric-reducing antioxidant power (FRAP), total thiols, nitrites, albumin, and total protein levels. DPPH was interpreted as an ex vivo index of serum radical-scavenging capacity rather than as a direct measure of in vivo oxidative damage. Participants were grouped according to vaccine regimen and prior SARS-CoV-2 infection status. In addition to evaluating overall and regimen-specific post-booster redox changes, we explored whether prior infection status and host-related factors, including age, sex, and comorbidity burden, modified the magnitude of these responses. We also performed supportive correlation, effect-size, and sensitivity analyses to further characterize the consistency of the observed pattern.

## 2. Results

### 2.1. Participant Characteristics

The study included 410 adult participants, of whom 232 (56.6%) were women, and 178 (43.4%) were men (Table 1). The mean age of the cohort was 52.1 ± 1.6 years (range: 18–82 years). Participants were grouped into four vaccine regimens: 88 individuals (21.5%) received three doses of the Pfizer-BioNTech vaccine (PPP), 156 (38.0%) received three doses of the Sinopharm [Vero Cell]-Inactivated vaccine (SSS), 33 (8.0%) received three doses of Sputnik V (SpSpSP), and 133 (32.4%) received a heterologous combination of two Sinopharm doses followed by a Pfizer-BioNTech booster (SSP). The average age was higher in the Sinopharm-based regimens (SSS and SSP, approximately 54 years) than in the Pfizer and Sputnik groups (49–52 years), reflecting the demographic pattern of the vaccine rollout in Serbia, although this difference did not reach statistical significance (one-way ANOVA, *p* = 0.08).

Prior SARS-CoV-2 infection was documented in 213 participants (51.9%), while 197 (48.1%) reported no history of infection (Table 1). The prevalence of previous COVID-19 infection varied across vaccine regimens, ranging from 37.5% in the PPP group to 64.1% in the SSS group. Hypertension was the most frequent comorbidity, reported in 91 participants (22.2%), followed by diabetes mellitus in 19 (4.6%) and thyroid disease in 14 (3.4%). Other chronic conditions were uncommon overall (13/410; 3.2%). Sex distribution and the prevalence of comorbidities were similar among vaccine groups, and no meaningful differences were observed in baseline biochemical or redox parameters. The mean interval between the second and third vaccine doses was 5.1 ± 0.4 months, with no significant variation among regimens. Overall, these findings indicate that the study population was broadly comparable across vaccine regimens, apart from minor, non-significant age differences.

### 2.2. Paired Before- and After-Booster Comparisons

**Figure 1:**
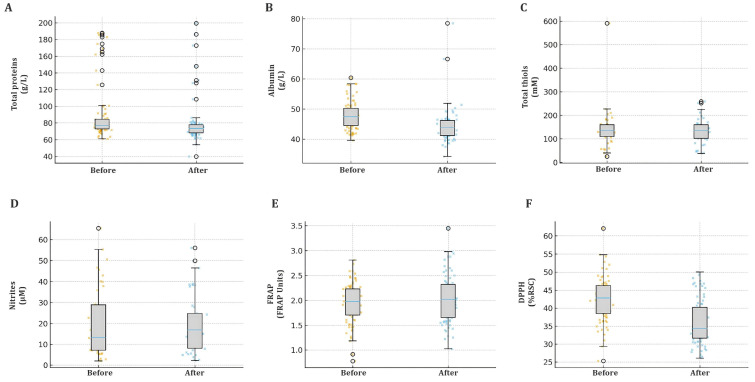
Before- and after-booster comparison of serum redox biomarkers in the total cohort (410 subjects), shown for (**A**) total proteins, (**B**) albumin, (**C**) total thiols, (**D**) nitrites, (**E**) ferric-reducing antioxidant power—FRAP, and (**F**) DPPH radical-scavenging activity. Boxes depict the median and interquartile range, whiskers indicate 1.5 × IQR, and outliers are shown as individual points. The 95% confidence intervals for mean paired differences were calculated from the paired Δ values using the t distribution and are reported in the text.

Paired analyses across all 410 study participants demonstrated measurable but generally modest shifts in serum redox parameters one month after administration of the COVID-19 booster (Figure 1). The most consistent finding was a significant reduction in DPPH radical-scavenging activity (mean Δ = −2.16%, 95% CI −2.96 to −1.36; *p* < 0.001), indicating a short-term decrease in DPPH-based radical-scavenging capacity. Albumin and total protein concentrations also showed small but statistically significant declines after the booster (mean Δ = −0.95 g/L and −0.49 g/L, respectively; both *p* < 0.001). These reductions indicate a measurable shift in the buffering capacity of albumin-associated circulating proteins. Because individual protein fractions, vascular distribution, hydration status, and albumin synthesis/catabolism were not measured, the finding should be described conservatively as a decrease in measured albumin and total serum protein, rather than as direct proof of redistribution among specific protein classes. In contrast, FRAP values remained essentially unchanged (mean Δ = −0.01 a.u.; *p* = 0.058), suggesting relative preservation of the ferric-reducing antioxidant pool. Nitrite levels were stable (mean Δ = +0.05 µM; *p* = 0.51), and total thiols exhibited only a mild, non-significant decrease (mean Δ = −0.01 mM; *p* = 0.082).

Taken together, the post-booster profile in the cohort is characterised by decreases in DPPH, albumin, and total proteins, while FRAP, nitrite, and thiol levels remain stable. This pattern suggests a transient shift in circulating antioxidant buffering accompanying physiological immune activation. However, because direct markers of lipid peroxidation, protein oxidation, or DNA oxidation were not assessed, the data should not be interpreted as fully excluding oxidative stress.

### 2.3. Between-Group Differences in Redox Response by Vaccine Regimen

**Figure 2:**
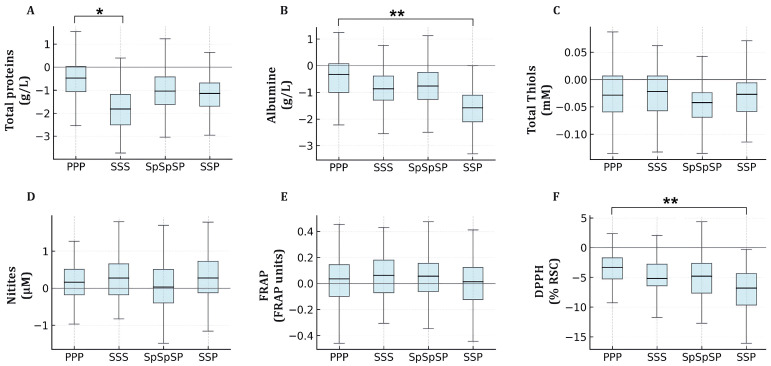
Between-group comparison of before-and-after changes (Δ) in serum redox biomarkers by vaccine regimen. Each panel shows Δ values for total proteins (**A**), albumin (**B**), total thiols (**C**), nitrites (**D**), FRAP (**E**), and DPPH radical-scavenging activity (**F**) across the four vaccine regimens—Pfizer-BioNTech (PPP), Sinopharm (SSS), Sputnik V (SpSpSP), and Sinopharm/Pfizer (SSP). Δ values are plotted relative to zero, which represents no change between post- and pre-vaccination measurements. Boxes show medians and interquartile ranges with whiskers at 1.5 × IQR; where mean estimates are provided in the text; 95% confidence intervals were calculated from the corresponding Δ distributions using the t distribution. *—*p* < 0.05, **—*p* < 0.01.

To evaluate whether the vaccine platform influenced post-booster redox responses, post–pre differences (Δ) for each biomarker were compared across the four vaccine regimens using one-way ANOVA followed by Tukey’s post hoc test (Figure 2). Significant between-group effects were observed for total proteins (ANOVA *p* = 0.021), albumin (*p* = 0.0027), and DPPH radical-scavenging activity (*p* = 0.010), whereas total thiols (*p* = 0.64), nitrites (*p* = 0.79), and FRAP (*p* = 0.93) did not differ significantly among regimens. The heterologous SSP combination showed the largest decrease in albumin (mean Δ = −1.49 ± 0.41 g/L; *p* = 0.002 vs. PPP) and DPPH activity (mean Δ = −6.17 ± 1.78%; *p* = 0.004 vs. PPP). The Sinopharm regimen produced a smaller but still significant reduction in total proteins (mean Δ = −1.57 ± 0.46 g/L; *p* = 0.014 vs. PPP). The PPP and SpSpSP groups showed comparable trends in DPPH decreases, although differences between them were not statistically significant (*p* > 0.05). Overall, these results indicate that Sinopharm-based regimens, especially the heterologous SSP sequence, were associated with more substantial transient reductions in measured circulating proteins and DPPH-based radical-scavenging activity than the homologous PPP regimen.

### 2.4. Stratified Analysis by Prior COVID-19 Status

**Figure 3:**
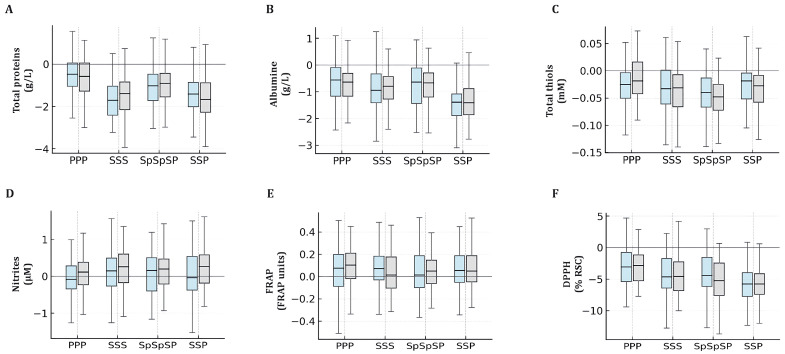
Stratified analysis of after–before changes (Δ) in serum redox biomarkers by prior COVID-19 status. Panels (A–F) display Δ values for total proteins (**A**), albumin (**B**), total thiols (**C**), nitrites (**D**), FRAP (**E**), and DPPH radical-scavenging activity (**F**) in participants with (gray) and without (light blue) previous SARS-CoV-2 infection. Boxes denote the interquartile range, medians are shown as black lines, and whiskers indicate 1.5 × IQR. Mean subgroup differences and their 95% confidence intervals were calculated from Δ values as mean ± t_0.975,n−1_ × SEM.

To assess whether prior SARS-CoV-2 infection affected post-booster redox responses, participants were stratified by prior COVID-19 status, and within-group Δ changes (after–before) in each biomarker were analyzed (Figure 3; next page). Across all vaccine regimens, the direction of change was largely consistent between previously infected (COVID-positive) and non-infected (COVID-negative) participants, indicating that prior viral exposure did not substantially alter the qualitative pattern of antioxidant modulation. The most pronounced effects again appeared in the heterologous SSP group, where both COVID-positive and COVID-negative subgroups showed significant decreases in albumin (mean Δ = −1.4 ± 0.4 g/L; *p* < 0.01) and DPPH activity (mean Δ = −5.9 ± 1.7%; *p* = 0.01). Participants vaccinated with Sinopharm only (SSS) showed a modest decline in total protein levels, regardless of prior infection (mean Δ ≈ −1.5 g/L; *p* < 0.05). No meaningful COVID-status differences were observed for FRAP, nitrites, or thiols in any vaccine group (two-way ANOVA interaction *p* > 0.2).

### 2.5. Associations of Age, Sex, and Comorbidities with Redox Parameter Changes

To assess independent predictors of post-booster redox modulation, multivariable linear regression models were constructed with ΔDPPH, Δalbumin, and ΔFRAP values as dependent variables, and age, sex, comorbidity status (binary), vaccine regimen, and prior COVID-19 infection as covariates (Table 2). The analysis showed that age and comorbidity presence were significant predictors of greater declines in both DPPH and albumin. In contrast, sex and prior infection did not have measurable effects after adjustment. Each additional year of age was associated with a −0.09% reduction in ΔDPPH (β = −0.09, 95% CI −0.16 to −0.03, *p* = 0.004) and a −0.03 g/L reduction in Δalbumin (β = −0.03, 95% CI −0.05 to −0.01, *p* = 0.006). Participants with at least one comorbidity (hypertension, diabetes, or thyroid disorder) showed more pronounced DPPH decreases (β = −1.7, 95% CI −2.9 to −0.4, *p* = 0.01) and albumin reductions (β = −0.6, 95% CI −1.0 to −0.2, *p* = 0.002) compared with those without comorbidities.

No significant predictors were identified for ΔFRAP, indicating that non-enzymatic ferric-reducing antioxidant potential remained largely stable across demographic strata. Overall, these findings indicate that older age and comorbidities accentuate the transient post-vaccination decrease in DPPH-based radical-scavenging capacity and albumin. In contrast, sex and infection history do not substantially modify the redox trajectory.

### 2.6. Correlation, Effect Size, and Internal Consistency Analyses

**Figure 4:**
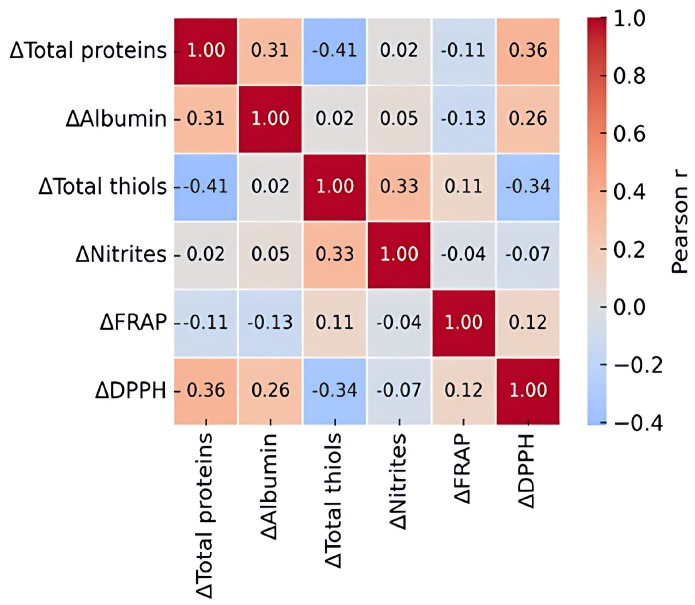
Correlation matrix of Δ (afterbefore) changes across serum redox biomarkers pooled from all vaccine groups.

**Figure 5:**
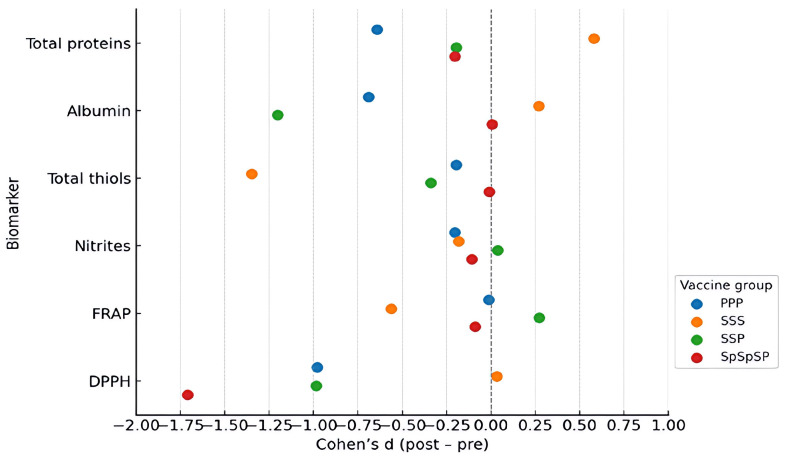
Effect sizes (Cohen’s d) across vaccine regimens for serum redox biomarkers. Forest plot showing within-group post-booster changes (Cohen’s d) for six serum biomarkers.

Correlation analysis of Δ values showed that changes in DPPH activity were positively associated with changes in total proteins and albumin, whereas FRAP remained largely uncorrelated with the other biomarkers (Figure 4). These findings support the interpretation that post-booster reductions in DPPH-based radical-scavenging capacity were linked mainly to shifts in circulating protein-associated antioxidant buffering, rather than to a uniform change across all antioxidant systems. Effect-size analysis further showed that DPPH exhibited the most consistent within-group decrease across vaccine regimens, while albumin showed smaller but still notable reductions, particularly in the SSP group. Between-group effect sizes (Figure 5) were modest overall, indicating that the principal pattern was shared across regimens despite quantitative differences.

### 2.7. Sensitivity Analyses

Sensitivity analyses confirmed the stability of the main findings. Nonparametric testing reproduced the same significance pattern as the primary analyses, and exclusion of extreme outliers or adjustment for the interval between the second and third vaccine doses did not materially change the results. Together, these analyses support the robustness of the observed short-term post-booster redox-associated response (Figure 5).

## 3. Discussion

This study analyzes systemic redox alterations one month after COVID-19 booster vaccination across four vaccine regimens. The findings indicate a reproducible biochemical pattern compatible with a transient shift in serum antioxidant buffering rather than a sustained redox disturbance. However, the interpretation should be made with caution. DPPH is an ex vivo chemical radical-scavenging assay and should be regarded as a surrogate of serum radical-scavenging capacity, not as a direct measure of in vivo redox status or oxidative tissue injury [18,19]. Moreover, the absence of significant changes in FRAP, nitrites, and total thiols does not fully exclude oxidative stress, because direct markers of oxidative damage, such as lipid peroxidation products, protein carbonylation, or oxidised DNA products, were not measured. Therefore, the data support a controlled and transient biochemical response, but not a definitive absence of oxidative stress.

The principal post-booster trend was a gradual but consistent decline in serum DPPH radical-scavenging capacity, accompanied by minor reductions in albumin and total protein levels. As DPPH primarily reflects the free-radical-scavenging, hydrogen-donor activity of accessible antioxidants, a reduction in DPPH most likely indicates temporary depletion or reduced availability of readily reactive antioxidant pools rather than permanent oxidative damage [19]. Because albumin is a major extracellular redox buffer and nucleophilic scavenger, the concurrent decline in albumin supports the interpretation that part of the diminished DPPH capacity may arise from changes in protein-associated antioxidant buffering [20]. At the same time, albumin behaves as a negative acute-phase protein during inflammatory signalling, and cytokine-driven acute-phase responses can reprioritise hepatic protein synthesis and alter circulating protein composition [17]. Since only total protein and albumin were quantified, our data should not be read as proof of redistribution among specific protein fractions; rather, they demonstrate a modest decrease in measured albumin and total serum protein that is biologically compatible with acute-phase regulation, vascular distributional changes, catabolism, or hydration-related influences.

These findings should also be positioned within the broader COVID-19 biomarker literature. COVID-19 progression and outcome are not captured by redox variables alone, but by integrated inflammatory, coagulation, and metabolic networks. For example, combined laboratory indices including lymphocyte/monocyte count, D-dimer, and iron status have been shown to improve prediction of COVID-19 course and outcome in vulnerable populations [15]. This is relevant for the present study because iron metabolism provides a mechanistic bridge between inflammation and redox biology: disturbed iron handling can promote ROS generation, lipid peroxidation, and ferroptosis-related pathways in severe SARS-CoV-2 infection [16]. The present redox panel therefore captures only one part of a wider systemic response and should be interpreted alongside, rather than instead of, inflammatory-cell, coagulation, acute-phase, and iron-related biomarkers. This broader framework is particularly important when translating biochemical redox findings into clinical meaning, because modest changes in antioxidant-capacity assays do not necessarily correspond to clinically relevant oxidative injury.

Comparative analyses showed that SSS-based regimens, particularly the heterologous SSP sequence, were associated with more pronounced short-term reductions in albumin and DPPH activity than the homologous PPP regimen. This pattern is biologically plausible, as mixed-platform boosting may induce a stronger short-term immunometabolic response, including cytokine activity and reactive oxygen species signalling during lymphocyte activation [21]. Nevertheless, vaccine allocation during the Serbian rollout was not random, and regimen groups differed modestly in age distribution, prior infection prevalence, and comorbidity structure. These factors may introduce residual confounding even after multivariable adjustment. The observed differences should therefore be interpreted as regimen-associated patterns rather than definitive causal evidence that one vaccine platform intrinsically produces a stronger redox response.

Stratified analyses by prior SARS-CoV-2 infection further showed that the qualitative pattern of post-booster redox change was largely consistent between previously infected and non-infected participants. Although the heterologous SSP regimen again showed the most pronounced decreases in albumin and DPPH activity, prior infection itself did not materially modify the overall response trajectory. This suggests that the observed post-booster antioxidant modulation was driven more strongly by booster regimen and host-related factors than by previous viral exposure.

Multivariable analyses further indicated that older age and the presence of comorbidities were associated with greater declines in DPPH activity and albumin after booster vaccination, whereas sex and prior COVID-19 status were not independently associated with these changes. These findings support the concept that reduced redox adaptability in older and comorbid individuals may amplify short-term antioxidant buffering changes during vaccine-induced immune activation [14,22]. Within this framework, the transient reductions observed in DPPH and albumin are most consistent with short-term consumption, reduced availability, or redistribution of antioxidant resources during immune activation, rather than structural oxidative injury.

Mechanistically, immune activation is accompanied by controlled production of reactive oxygen species as signalling intermediates and effector molecules. ROS participate in antigen-receptor signalling, phagocyte respiratory burst, cytokine-dependent immune responses, and T-cell activation; at physiological levels, these processes are not merely damaging but are required for immune regulation [21]. During booster-induced antigenic stimulation, a temporary increase in ROS/RNS signalling could consume readily available serum antioxidants. In parallel, inflammatory mediators may affect hepatic protein synthesis and the circulating pool of negative acute-phase proteins such as albumin [17]. Together, these processes provide a plausible explanation for a temporary decrease in albumin-linked and DPPH-measured antioxidant capacity while FRAP, nitrites, and total thiols remain relatively stable.

Several limitations should be explicitly considered. First, the study did not include iron-related variables such as serum iron, ferritin, transferrin saturation, transferrin, or hepcidin, despite the strong biological connection between iron handling, oxidative stress, ferroptosis, and COVID-19 pathophysiology [15,16]. Second, inflammatory-cell counts, D-dimer, CRP, fibrinogen, and other coagulation or acute-phase markers were not integrated with the redox panel. Third, lifestyle and nutritional variables that can influence antioxidant capacity, including diet, micronutrient intake, antioxidant supplement use, smoking status, recent exercise, hydration, alcohol intake, and recent medication exposure, were not comprehensively captured; this is particularly relevant for DPPH, which reflects ex vivo radical-scavenging capacity and may be affected by circulating dietary or nutritional antioxidants. Fourth, no direct oxidative-damage biomarkers were measured, and only one post-booster time point was available. Finally, the observational design and heterogeneity of vaccine allocation, age, comorbidities, and prior infection history may have introduced residual confounding that cannot be fully eliminated by regression models. Vaccine assignment followed real-world national rollout patterns rather than random allocation, so unmeasured differences in health status, risk perception, access to vaccination, or clinical indication may have influenced regimen-specific comparisons. These limitations reduce the ability to infer clinical consequences from the biochemical changes and underscore that the results should be regarded as hypothesis-generating.

In summary, COVID-19 booster vaccination was associated with a reproducible yet modest and apparently controlled modulation of circulating antioxidant markers. The transient decline in DPPH-measured and albumin-linked antioxidant capacity most likely reflects adaptive metabolic engagement of redox systems during immune activation. These findings contribute to understanding the redox dimension of vaccine responses, but their translational relevance will require future studies that combine redox markers with inflammatory, coagulation, iron-metabolism, nutritional, and clinical outcome data.

## 4. Materials and Methods

### 4.1. Study Design and Participants

This longitudinal cohort study included 410 adult participants (≥18 years) who had received three doses of COVID-19 vaccines in accordance with national immunization guidelines in Serbia. Participants were categorized according to vaccine type (Pfizer-BioNTech (BNT162b2), Sinopharm [Vero Cell]-Inactivated, Sputnik V, or heterologous Sinopharm/Pfizer-BioNTech) and prior SARS-CoV-2 infection status. Exclusion criteria included participants younger than 18 years and individuals who had not been vaccinated against SARS-CoV-2. All participants included in the study voluntarily signed an informed consent form, completed a self-questionnaire (Appendix A), and provided blood samples for laboratory analysis.

### 4.2. Sample Collection and Processing

Venous blood (4–6 mL) was collected from the antecubital vein using a vacuum collection system into serum tubes without anticoagulant (VACUETTE^®^, Greiner Bio-One GmbH, Kremsmünster, Austria). After clotting at room temperature for 15–30 min, the sera were centrifuged at 3000 rpm for 10 min (Gyrozen 416, Seoul, South Korea) to separate the serum. Serum aliquots were stored at −80 °C until analysis.

### 4.3. Biochemical Assays

Serum total protein and albumin were measured spectrophotometrically on an automated biochemical analyzer BA200 (BioSystems S.A., Barcelona, Spain) using the biuret and bromocresol-green methods, respectively [23,24]. Total thiols were quantified by Ellman’s method using DTNB reagent [25,26], with absorbance recorded at 412 nm. Nitrite levels, as an indicator of nitric oxide production, were determined using the Griess assay [27]. Antioxidant activity was assessed using the DPPH radical-scavenging assay [28] and the FRAP assay [29]. For the DPPH assay, samples were incubated with 2,2-diphenyl-1-picrylhydrazyl radicals (DPPH^•^), and the decrease in absorbance of the stable purple radical was measured at 515 nm. Radical-scavenging capacity was calculated relative to reagent blanks and expressed as percentage inhibition. DPPH was interpreted as an ex vivo measure of serum radical-scavenging capacity and not as a direct marker of in vivo oxidative damage. In the FRAP assay, the reduction of ferric (Fe^3+^) to ferrous (Fe^2+^) ions in the presence of TPTZ (2,4,6-Tris(2-pyridyl)-s-triazine) under acidic conditions was quantified by measuring absorbance at 593 nm. Concentrations were calculated from a FeSO_4_ calibration curve and expressed in FRAP units. Absorbance was recorded using a Multiskan SkyHigh microplate spectrophotometer (Thermo Fisher Scientific, Vantaa, Finland).

### 4.4. Statistical Analysis

Statistical analyses were performed using custom scripts written in Python 3.14.0 and executed in Visual Studio Code version 1.116, using the pandas 2.3.3, NumPy 2.3.5, SciPy 1.16.3, and statsmodels 0.14.5 libraries. Continuous variables are presented as mean ± SEM for descriptive and raw pre/post values, and changes are summarized as mean Δ with 95% confidence intervals where applicable. For paired comparisons in Figure 1, 95% confidence intervals for the mean paired difference were calculated as mean Δ ± t_0.975,n−1_ × SEMΔ. For groupwise Δ comparisons in Figure 2 and Figure 3, boxplots display medians, interquartile ranges, and whiskers; the boxplots themselves do not display standalone confidence-interval bars. When mean subgroup estimates were reported in the text, 95% confidence intervals were calculated from the corresponding Δ distributions using the same t-based approach. Normality was assessed using the Shapiro–Wilk test before selection of the appropriate statistical procedure. Within-group pre/post comparisons were performed using paired t-tests or Wilcoxon signed-rank tests, as appropriate. Between-group comparisons of Δ (post–pre) values were assessed using one-way ANOVA with Tukey’s post hoc test or the Kruskal–Wallis test, as appropriate. Stratified analyses were performed by prior SARS-CoV-2 infection status, and interaction testing was used to assess whether infection history modified post-booster responses across vaccine regimens. Multivariable linear regression models were constructed for ΔDPPH, Δalbumin, and ΔFRAP using age, sex, comorbidity status, vaccine regimen, and prior COVID-19 infection as covariates. Pairwise Pearson correlation coefficients were calculated among Δ biomarker values to explore coordinated redox responses. Effect sizes were estimated using Cohen’s d for within-group pre/post changes and partial η^2^ for between-group comparisons. Sensitivity analyses included nonparametric confirmation of the primary results, exclusion of extreme outliers, and adjustment for the interval between the second and third vaccine doses. Statistical significance was set at *p* < 0.05.

## 5. Conclusions

In conclusion, COVID-19 booster vaccination induced modest short-term changes in circulating antioxidant-related markers, primarily reflected by decreased DPPH-based radical-scavenging activity and slight reductions in albumin and total protein, while FRAP, nitrite, and total thiol levels remained stable. These effects were most evident in Sinopharm-based and heterologous regimens. Prior SARS-CoV-2 infection did not substantially alter the qualitative response pattern, whereas older age and comorbidity burden were associated with greater short-term declines in DPPH activity and albumin. Overall, the findings support a reproducible but controlled shift in serum antioxidant buffering associated with booster-induced immune activation. They should not be interpreted as proof of the absence of oxidative stress or as direct evidence of clinically relevant oxidative injury, because DPPH is a surrogate assay, and direct oxidative damage, iron metabolism, inflammatory cells, coagulation, lifestyle, and nutritional variables were not assessed. Future studies should integrate redox markers with broader clinical and immunometabolic biomarker panels to define the translational significance of these changes.

## Figures and Tables

**Table 1:** Baseline characteristics of participants by vaccine regimen and prior COVID-19 status.

	Overall (*n* = 410)	PPP (3 × Pfizer) (*n* = 88)	SSS (3 × Sinopharm) (*n* = 156)	SpSpSP (3 × Sputnik V) (*n* = 33)	SSP (Sinopharm × 2 + Pfizer) (*n* = 133)
Age (mean ± SEM, yrs)	52.1 ± 1.6	49 ± 2	54 ± 1	53 ± 2	52 ± 1
Female sex (%)	232 (56.6)	52 (59.1)	88 (56.4)	18 (54.5)	74 (55.6)
Male sex (%)	178 (43.4)	36 (40.9)	68 (43.6%)	15 (45.5%)	59 (44.4%)
Prior COVID-19 infection (%)	213 (51.9)	33 (37.5)	100 (64.1)	19 (57.6)	61 (45.9)
COVID-negative (%)	197 (48.1)	55 (62.5)	56 (35.9)	14 (42.4)	72 (54.1)
Hypertension (%)	91 (22.2)	17 (19.3)	42 (26.9)	7 (21.2)	25 (18.8)
Diabetes mellitus (%)	19 (4.6)	4 (4.5)	8 (5.1)	2 (6.1)	5 (3.8)
Thyroid disease (%)	14 (3.4)	3 (3.4)	5 (3.2)	1 (3.0)	5 (3.8)
Other chronic conditions (%)	13 (3.2)	2 (2.3)	6 (3.8)	1 (3.0)	4 (3.0)
Interval 2nd→3rd dose (months)	5.1 ± 0.4	5.0 ± 0.5	5.3 ± 0.4	5.0 ± 0.6	5.2 ± 0.5

Values are presented as mean ± standard error of the mean (SEM) or number (percentage). No significant baseline differences were observed in sex distribution or comorbidity frequency among groups; age differences approached but did not reach significance (ANOVA, *p* = 0.08).

**Table 2:** Multivariable regression models for predictors of ΔDPPH, ΔAlbumin, and ΔFRAP.

Predictor	ΔDPPH (% RSC) β (95% CI)	*p*-Value	ΔALBUMIN (G/L) β (95% CI)	*p*-Value	ΔFRAP (FRAP Units) β (95% CI)	*p*-Value
Age (years)	−0.09 (−0.16 to −0.03)	**0.004**	−0.03 (−0.05 to −0.01)	**0.006**	+0.001 (−0.004 to 0.005)	0.72
Sex (male)	+0.6 (−1.1 to +2.3)	0.49	+0.1 (−0.3 to +0.5)	0.63	−0.002 (−0.015 to +0.011)	0.76
Comorbidity (yes)	−1.7 (−2.9 to −0.4)	**0.01**	−0.6 (−1.0 to −0.2)	**0.002**	+0.008 (−0.011 to +0.027)	0.42
**Vaccine regimen**						
SSS	−0.9 (−2.3 to +0.5)	0.20	−0.2 (−0.6 to +0.3)	0.47	+0.005 (−0.010 to +0.020)	0.52
SpSpSP	−1.3 (−3.1 to +0.6)	0.18	−0.4 (−0.8 to +0.1)	0.11	+0.004 (−0.013 to +0.021)	0.61
SSP	−2.8 (−4.1 to −1.5)	<0.001	−0.9 (−1.3 to −0.5)	**<0.001**	+0.006 (−0.009 to +0.021)	0.46
Prior COVID-19 (yes)	−0.5 (−1.8 to +0.7)	0.41	−0.1 (−0.4 to +0.3)	0.69	+0.002 (−0.011 to +0.015)	0.76
Intercept	−2.2 (−3.9 to −0.5)	**0.01**	−0.7 (−1.1 to −0.2)	**0.004**	+0.012 (−0.006 to +0.030)	0.18

RSC = Radical Scavenging Capacity; β = standardized regression coefficient; CI = confidence interval. Significant predictors (*p* < 0.05) are highlighted in bold.

## Data Availability

The data presented in this study are openly available in the Digital Repository of Archived Publications of the Institute for Biological Research “Siniša Stanković” at https://hdl.handle.net/21.15107/rcub_ibiss_8111 accessed on 17 May 2026.

## Appendix A. Participant Self-Questionnaire

All participants who gave their informed consent voluntarily filled out a self-questionnaire with the following inquiries:

- History of previous SARS-CoV-2 infections with the date on which the symptoms appeared or the date of the last positive PCR result;
- History of vaccination (date and name of the 1st, 2nd and 3rd dose of SARS-CoV-2 vaccine);
- Diseases of the cardiovascular system (hypertension, ischemic heart disease, heart failure, heart valve diseases, myocarditis, endocarditis, pericarditis, deep vein thrombosis, etc.);
- Diseases of the endocrine system (diabetes mellitus, metabolic syndrome, hyperthyroidism, hypothyroidism, Cushing’s syndrome, etc.);
- Diseases of the nervous system (cerebrovascular diseases, stroke, epilepsy, multiple sclerosis, polyneuropathy, neuroborreliosis, etc.);
- Liver diseases (hepatitis B, hepatitis C, cirrhosis, etc.);
- Autoimmune diseases (systemic lupus, rheumatoid arthritis, systemic sclerosis, etc.);
- Pulmonary diseases (asthma, COPD, emphysema, pulmonary hypertension, etc.);
- Kidney diseases (hypertensive nephropathy, diabetic nephropathy, hydronephrosis, chronic renal insufficiency, etc.);
- The presence of allergic reactions (atopy, allergic dermatitis, allergic rhinitis, allergic asthma, etc.);
- Primary and secondary immunodeficiencies (yes or no, and which);
- Severe diseases of the hematopoietic system (yes or no, and which);
- Oncological diseases (yes or no, and which);
- Pregnancy and breastfeeding status.
